# Early post-operative nerve complications after primary open Latarjet: a single-surgeon series of 164 shoulders

**DOI:** 10.1016/j.jseint.2026.101710

**Published:** 2026-04-10

**Authors:** Alexander J. Vervaecke, Bradley Schoch, Filip Cosic, Eduard Van Eecke, Jean-David Werthel

**Affiliations:** a Hôpital Ambroise-Paré, Boulogne Billancourt, France; b Orthopaedic Center Antwerp (Orthoca), Antwerp, Belgium; c Monica Orthopaedic Research (MoRe) foundation, Antwerp, Belgium; d Department of Orthopaedic Surgery, Mayo Clinic, Jacksonville, FL, USA; e Department of Orthopaedic Surgery, Austin Health, Victoria, Australia; f Department of Orthopaedic Surgery, Roeselare, Belgium

**Keywords:** Shoulder instability, Shoulder surgery, Anterior dislocation, Latarjet, Bone block, Nerve palsy, Neurapraxia, Surgical complications

## Abstract

**Background:**

Post-operative nerve complications remain a concern following the Latarjet procedure due to the proximity of the musculocutaneous, axillary, and suprascapular nerves during key surgical steps. Reported incidence rates of nerve injuries with this procedure vary considerably in the literature. The aim of this study was to evaluate the incidence of early post-operative nerve complications in consecutive patients undergoing a primary open Latarjet procedure treated with a standardized surgical technique.

**Method:**

A single-center, single-surgeon retrospective case series of patients undergoing primary open Latarjet procedure between January 2019 and June 2025 was conducted. A total of 176 consecutive patients were identified, with 93.3% follow-up. All procedures were performed using a standard deltopectoral approach with a subscapularis split limited to the muscular portion and a vertical capsulotomy. Routine follow-up was conducted at four weeks, three months, and six months post-operatively with the senior surgeon. Medical records were reviewed for documentation of post-operative complications, neurological symptoms, referrals to a neurologist, performance of electromyography, and emergency department evaluations within the first six months after surgery.

**Results:**

Among the 164 included patients, 137 (83.5%) were male. The right shoulder was operated in 92 cases (56.1%), and the mean age at surgery was 27.4 ± 8.2 years (range, 16.4-69.0). Post-operatively, one patient (0.6%) developed a transient sensory neurapraxia of the median nerve, presenting as hypesthesia and paresthesia on the volar aspect of the thumb, which resolved by six months. No musculocutaneous, axillary, or suprascapular nerve deficits were identified, and no patient required surgical exploration or neurolysis. Two patients (1.2%) developed an acute surgical site infection requiring washout, débridement, and screw retention. One patient (0.6%) experienced a superficial venous thrombosis of the cephalic and basilic veins, managed with oral anticoagulation. One patient (0.6%) sustained a recurrent anterior dislocation following a new-onset epileptic seizure, treated conservatively. No additional clinical complications were documented. The overall early clinical complication rate was 3.0% (n = 5), and the reoperation rate within six months was 1.2% (n = 2).

**Conclusion:**

In this single-surgeon consecutive series of primary open Latarjet procedures, early post-operative nerve complications were infrequent, and the overall clinical complication rate was low. These findings reflect outcomes achieved within a standardized surgical setting and should be interpreted in the context of surgeon experience and volume.

The Latarjet procedure is a well-established surgical option in the management of anterior shoulder instability, with very low failure rates reported at long-term follow-up.^14^ Its use, particularly in patients with bone loss, is widely accepted; however, there is considerable variation of indications and outcomes highlighted by regional differences in management. Specifically in the United States of America, the Latarjet is often reserved for revision or complex cases, whereas in France it is commonly performed as a primary stabilization procedure regardless of bone loss.^2^^,^^19^ Despite the increasing popularity of the Latarjet procedure, concerns remain regarding associated complication rates, including post-operative neurological complications, which have been reported to be as high as 18.4%.^15^^,^^24^ In approximately 5% of cases, these nerve injuries have been reported to be permanent.^24^

Neurological complications following the Latarjet procedure are a concern due to the proximity of the musculocutaneous, axillary, and suprascapular nerves to the surgical field throughout the procedure, with significant variations in distances occurring secondary to limb position and patient anatomy.^5^^,^^17^^,^^21^ While the majority of neurological injuries that occur during Latarjet are transitory,^10^^,^^23^ residual nerve palsies are potentially devastating complications and often require further surgical management by a brachial plexus/nerve specialist.^11^ Thus, efforts to improve surgical techniques to minimize neurological injury are critical to ensure optimal patient outcomes following Latarjet. Previous work has been conducted using neuromonitoring, which identified the musculocutaneous and axillary nerves to be at particular risk during glenoid exposure and graft insertion.^7^ Subsequent alterations to the surgical technique to minimize nerve stretch, focusing on arm and retractor position, graft location, and operative time, have demonstrated promising results in the reduction of neurological complications.^24^

This study aimed to report the incidence of postoperative nerve complications in a consecutive series of patients undergoing a primary Latarjet procedure utilizing a standardized surgical technique focused on minimizing the risk of neurological injury. We hypothesized that the incidence of neurological complications would be low in primary Latarjet cases when using this approach.

## Method

### Study design

We conducted a single-center, retrospective case series including all patients who underwent a primary open Latarjet procedure between January 2019 and June 2025. All surgeries were performed using a standardized technique by the senior author (JDW), a fellowship-trained shoulder surgeon with >5 years of independent practice and extensive exposure to shoulder instability pathology. In his practice, the Latarjet procedure represents the primary operative treatment for anterior shoulder instability, with surgical indications based on an Instability Severity Index Score ≥ 2. On average, more than 25 Latarjet procedures are performed annually. Patients were identified through the institutional electronic medical record system by cross-referencing the procedural code for the Latarjet procedure with the surgeon identifier. Each case was manually verified by reviewing the operative report and both pre-operative and post-operative documentation to confirm accurate coding and inclusion.

Exclusion criteria were: (1) prior open shoulder surgery (eg, open Bankart repair or capsular shift), (2) concomitant treatment of Hill–Sachs lesions (eg, remplissage or bone grafting), and (3) age <16 years at the time of surgery. A total of 176 patients met the initial criteria. Those without any post-operative documentation (no clinical note or post-operative imaging within 6 months) were considered lost to follow-up and excluded (n = 12; 6.7%), leaving 164 patients for analysis. Patient charts were retrospectively reviewed to identify the primary outcome, defined as any post-operative nerve complication occurring within 6 months. Secondary outcomes included overall complication rate and reoperation within the same timeframe. Institutional review board approval was obtained for this retrospective chart review (Réference 19-2025).

### Surgical procedure

All procedures were performed under combined general and locoregional (single-shot interscalene block) anesthesia, with the patient in the supine position and a small rolled towel placed between the scapulae to achieve slight scapular retraction.^21^ The arm rested alongside the body without arm support to allow unrestricted manipulation. A 4.5-cm deltopectoral incision was made from the tip of the coracoid toward the axillary fold. The deltopectoral interval was identified and developed. The cephalic vein was retracted laterally. After exposure of the coracoid, the coracoacromial ligament (CAL) was identified, and the clavipectoral fascia was incised horizontally just distal to and in line with the CAL and then extended vertically along the lateral edge of the conjoint tendon. Blunt dissection was carried out underneath the conjoint tendon.

A valve retractor was then positioned, and the arm was externally rotated to facilitate detachment of the CAL from the lateral aspect of the coracoid. The arm was then repositioned into internal rotation, and the pectoralis minor was detached from the medial aspect of the coracoid. Afterward, a gauze was carefully passed beneath the coracoid from medial to lateral to bluntly complete the medial release and to temporarily protect the brachial plexus during osteotomy. The coracoid was osteotomized from medial to lateral using a 90° oscillating saw. The graft was then mobilized, and under slight tension, residual soft tissue attachments were released laterally under direct visualization. Medially, blunt dissection using a sterile gauze and a fingertip was used to further release the posterior aspect of the conjoint tendon under slight traction of the bone block. The undersurface of the graft was decorticated using the saw, and two 4.5 mm parallel holes were drilled using the dedicated guide (Arthrex, Naples, FL, USA). A tag suture was placed through the superior hole of the bone block to facilitate retrieval of the graft. The coracoid was positioned medially under the valve retractor, and the arm was externally rotated to expose the subscapularis.

A Trillat retractor was placed inferiorly to the subscapularis muscle to identify the “3 sisters” vessels, confirming the level of the axillary nerve medially. The subscapularis was then split in line with its fibers within the muscular portion, typically at the junction between the inferior and middle thirds, or within the middle one-third in hyperlax patients, and bluntly separated from the capsule. A blunt Gelpi retractor was inserted into the split, and a gauze with retrieval suture was advanced along the scapular neck up to bluntly detach the subscapularis from the subscapularis fossa underneath. A Batman retractor was positioned medially along the scapular neck, and subsequently, a vertical capsulotomy was performed in line with the joint line to expose the glenoid. A small Fukuda retractor was positioned intra-articularly, and the anterior labrum and capsule below the equator were resected. A sharp Hohmann retractor was placed inferiorly under the glenoid neck to clearly visualize the 6'o clock position to guide proper positioning of the bone block and to protect the axillary nerve inferiorly. The glenoid rim was decorticated with a curved osteotome. Subsequently, the coracoid bone block was positioned flush with the glenoid rim using the traditional (non–congruent arc) technique at the subequatorial level and fixed with two 4.5 mm cannulated bicortical screws (Arthrex, Naples, FL, USA), oriented parallel to the joint line while avoiding lateral overhang of the graft. The joint was irrigated; 1 g of vancomycin powder was administered to the wound, and the deltopectoral interval was closed without capsular or subscapularis repair. Post-operatively, patients were immobilized in a simple sling for three weeks, with passive and active assisted range of motion initiated immediately.

**Figure 1:**
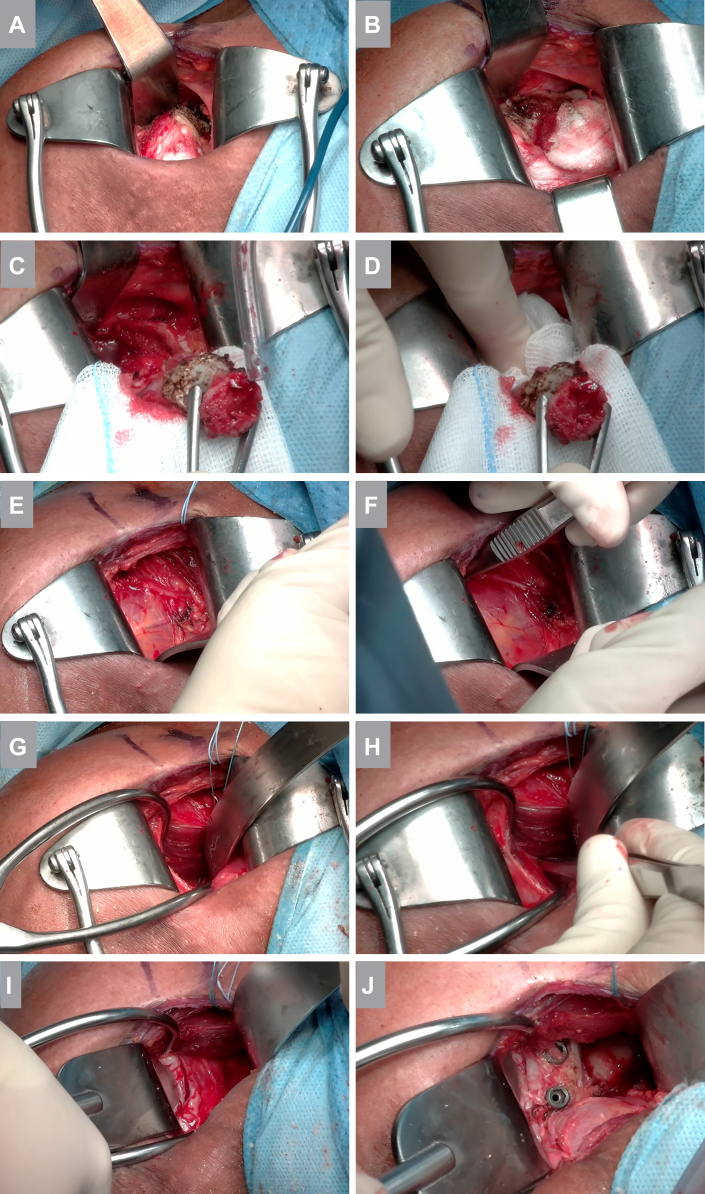
Intraoperative imaging of key nerve-protecting steps utilized during the open Latarjet procedure. (**A** and **B**) After release of the pectoralis minor, a sterile gauze is passed from medial to the coracoid and positioned underneath it using a rugine. This maneuver bluntly completes the pectoralis minor release and protects the underlying brachial plexus during the coracoid osteotomy. (**C** and **D**) Following completion of the osteotomy, excessive traction on the coracoid graft is avoided to prevent neurapraxia of the musculocutaneous nerve. The posteromedial release of the conjoint tendon is performed digitally using a wet gauze swab. The musculocutaneous nerve is not routinely identified. (**E** and **F**) A Trillat retractor is gently placed inferior to the subscapularis while identifying the level of the “3 sisters,” thereby estimating the medial height of the axillary nerve. Forceps positioned along the superior border of the subscapularis provide a reference to determine the appropriate level of the subscapularis split (junction of the middle/lower third, or the midportion in hyperlax patients). (**G** and **H**) Adequate exposure is obtained before capsulotomy. A Gelpi retractor is positioned in the muscular subscapularis split, and a gauze with retrieval suture is advanced along the scapular neck to the subscapularis fossa. A Batman retractor is positioned medially. The anterior joint line is palpated, the capsule lifted with forceps, and the capsulotomy performed under direct visualization. (**I**) Exposure for graft positioning: a Fukuda retractor is placed intra-articularly and a large Batman retractor medially. A sharp Hohmann retractor (not shown) is positioned inferior to the glenoid neck to clearly visualize the 6-o'clock region and protect the axillary nerve inferiorly. (**J**) Final construct demonstrating flush positioning of the coracoid bone block along the glenoid rim with screw orientation parallel to the joint line.

**Figure 2:**
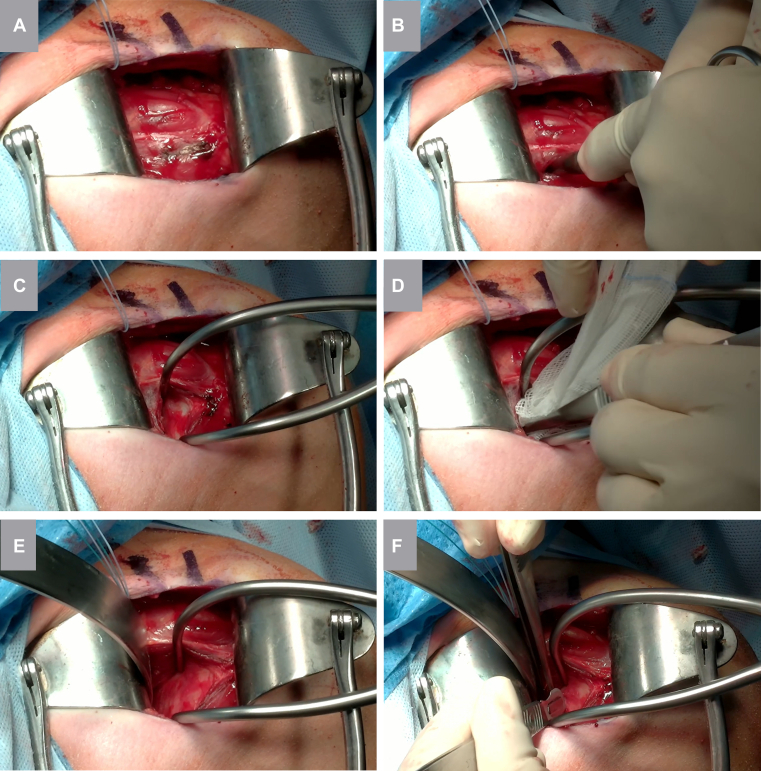
Detailed overview of the subscapularis split technique. (**A**) The planned height of the muscular split is marked with electrocautery, staying medial to the tendinous portion. (**B**) Mayo scissors are used to bluntly detach the subscapularis muscle fibers from the underlying anterior capsule. (**C**) A Gelpi retractor is positioned within the muscular split to maintain exposure. (**D**) A gauze swab (with retrieval suture) is advanced deep to the subscapularis muscle toward the fossa. (**E**) A large Batman retractor is placed medially over the anterior glenoid rim for improved exposure. (**F**) The joint line is palpated, the anterior capsule is elevated with forceps, and a vertical capsulotomy is performed from inferior to superior to create adequate space for subsequent Fukuda retractor placement.

In summary, the key nerve-protective steps employed, without routine direct nerve visualization (Fig. 1), were as follows: (1) placement of a sterile gauze swab medial and inferior to the coracoid during osteotomy to shield the brachial plexus; (2) avoidance of excessive traction on the coracoid process during graft mobilization to prevent musculocutaneous nerve neurapraxia; (3) completion of the posteromedial release by blunt digital dissection after osteotomy and graft mobilization; (4) identification of the inferior border of the subscapularis and the “3 sisters” vessels to determine the level of the axillary nerve; (5) standardized retractor positioning and ensuring adequate exposure before performing the capsulotomy (Fig. 2); and (6) orientation of screw trajectories parallel to the joint line to prevent posteromedial conflict with the suprascapular nerve distal to the second cortex.

### Patient evaluation

Routine follow-up visits were scheduled at 4 weeks, 3 months, and 6 months post-operatively with the senior surgeon (JDW), who reviewed every patient. At each visit, a structured clinical assessment was performed. Patients were considered lost to follow-up if no post-operative consultation was recorded within six months of surgery. A comprehensive review of the electronic medical records was conducted across the institution and its affiliated hospital network to capture any post-operative events including: (1) documentation of neurological symptoms such as paresthesia, hypesthesia, motor weakness; (2) referrals to neurologist; (3) electromyography evaluations; (4) emergency department visits related to the operated shoulders; and (5) surgical interventions. Neurological complications were defined as any new sensory or motor deficit recorded within 6 months of surgery. This included both transient and persistent deficits, such as sensory disturbances and motor weakness. No minimum duration of symptoms was required for classification, and both transient neurapraxias and persistent deficits were included when clinically documented.

### Statistical analysis

Data were analyzed descriptively. Continuous variables are presented as mean ± standard deviation and range, and categorical variables as counts and percentages. Exact binomial 95% confidence intervals (CIs; Clopper–Pearson method) were calculated for all reported proportions to account for the low number of events. No comparative statistics were performed given the study design.

## Results

### Patient demographics

In the 164 patients included, the mean age at the time of surgery was 27.4 ± 8.2 years (range, 16.4-69.0). The cohort included 137 males (83.5%) and 27 females (16.5%). The right shoulder was operated in 92 cases (56.1%) and the left shoulder in 72 (43.9%). Most patients had a history of recurrent anterior shoulder dislocation (multiple dislocations in 144 cases, 87.8%; single dislocation in 9 cases, 5.5%). Regarding activity profile, 139 patients (84.8%) reported regular sports participation; impact or contact sports were reported by 56 patients (34.1%), while 55 (33.5%) demonstrated clinical hyperlaxity on pre-operative evaluation.

### Post-operative complications

Within six months post-operatively, clinical complications were identified in 5 patients (3.0%). One patient (0.6%) developed a transient sensory neurapraxia affecting the median nerve, presenting as paresthesia and hypesthesia on the volar side of the thumb. Symptoms resolved completely within six months. No musculocutaneous, axillary, or suprascapular motor deficits were observed, and no patient required surgical exploration or neurolysis.

Two patients (1.2%) developed an acute post-operative Surgical Site Infection treated with washout, débridement, antibiotics, and screw retention. Both cases resolved without recurrent infection. One patient (0.6%) experienced a superficial venous thrombosis involving the cephalic and basilic veins, treated successfully with oral anticoagulation (apixaban 10 mg orally twice daily for 7 days followed by 5 mg twice daily for eleven weeks). One patient (0.6%) sustained a recurrent anterior dislocation following a new-onset epileptic seizure. The event was treated conservatively without surgical revision.

The overall reoperation rate within six months was 1.2% (2/164; 95% CI, 0.2-4.3%), and the overall surgery-related clinical complication rate was 3.0% (5/164; 95% CI, 1.0-6.9%). The overall nerve-related complication rate was 0.6% (1/164; 95% CI, 0.0-3.4%).

## Discussion

The Latarjet procedure demonstrates excellent clinical outcomes in the treatment of anterior shoulder instability, with recurrence rates consistently shown to be lower than that of isolated arthroscopic Bankart repair.^8^^,^^13^^,^^25^ However, complication rates following the Latarjet procedure are not insignificant, with rates as high as 30% previously published.^10^ Of concern is the high rate of neurological injury previously reported following Latarjet surgery, leading, in some cases, to permanent persistent neurological deficits.^10^^,^^23^^,^^24^ This study demonstrates that primary Latarjet procedures can be performed with a low risk of neurological injury, with only one case of transient neurapraxia occurring in a series of 164 patients. Furthermore, this series reported a low overall clinical complication rate of 3% and reoperation rate of 1.2% in the first six months post-operatively.

Prior studies have demonstrated highly variable rates of neurological injury following Latarjet procedures.^6^^,^^9^^,^^12^^,^^22^, ^23^, ^24^ The most commonly affected nerves are the musculocutaneous nerve and axillary nerve, although multiple other nerve injuries have been reported, including trunk-level plexopathies, radial nerve, and suprascapular nerve injuries.^10^^,^^18^ In our series, a single neurological complication was observed, consisting of a transient sensory neuropraxia affecting the volar aspect of the thumb, which resolved within six months post-operatively. Importantly, all procedures were performed under interscalene regional anesthesia. The distribution of symptoms observed in this case is consistent with sensory disturbances described following interscalene block at the C5-C6 nerve root level, which may manifest along the median nerve distribution. A causal relationship with the surgical procedure therefore cannot be definitively established, as regional anesthesia represents a potential confounding factor.^4^ Our findings contrast prior studies where rates of neurological injury are reported as high as 18.4%. An early paper in a series of 47 patients reported a neurological complication rate of 10%, involving the axillary, musculocutaneous, or radial nerves.^23^ In that series, the radial and musculocutaneous nerve injuries resolved; however, the axillary nerve dysfunction persisted. A more recent series demonstrated an 18.4% neurological complication rate in 38 patients, with 5.3% of patients demonstrating permanent motor dysfunction.^24^ The reason for the large discrepancy in neurological injury rates between our series and previously published work remains unclear. Possible explanations for the lower rate of neurological complications observed in this series include the use of a standardized surgical technique and the fact that the Latarjet procedure represents the primary operative treatment for anterior shoulder instability in the senior author's practice, leading to consistent procedural exposure and operative efficiency. In addition, this cohort exclusively included patients without prior open shoulder surgery. Although now less common, previous open capsular shift procedures may alter local anatomy through scarring and soft-tissue adhesions, potentially increasing technical complexity and the risk of neurological injury.

The surgical technique employed in this series follows a standardized approach and incorporates several steps aimed at minimizing the risk of nerve injury during critical phases of the procedure. Multiple technical modifications designed to reduce the neurological risk during Latarjet have previously been published and may aid in lowering neurological complications.^7^^,^^16^^,^^20^^,^^24^ Increased operative time, particularly increased time during key stages of the procedure where there is an increased risk to nerves, has been associated with neurological injury.^7^ Therefore, maximizing operative efficiency and minimization of prolonged retraction time are considered important. While precise operative time data were not systematically recorded for research purposes, the average operative duration in the senior author's practice is approximately 45 minutes from skin incision to closure. While this value should be interpreted cautiously, operative efficiency and minimization of sustained retraction may contribute to limiting nerve irritation.

A nerve stretch–reduction protocol incorporating specific limb positioning, graft handling, and minimization of sustained retraction has been described in conjunction with intraoperative neuromonitoring. This approach was associated with a reduction in intraoperative nerve alert events, although it did not demonstrate a statistically significant decrease in clinically evident post-operative neurological complications.^24^

While attention is commonly directed toward the axillary and musculocutaneous nerve, awareness of the suprascapular nerve during posterior wire and screw placement is also essential. A cadaveric study demonstrated a safe zone for screw placement within 10° of the face of the glenoid in the axial plane to avoid injury to the suprascapular nerve.^16^ Recognition of these at-risk stages, combined with careful surgical technique, may reduce the risk of neurological complications.

In addition to low rates of clinically documented neurological complications, the overall early complication and reoperation rates were also low. Two patients developed post-operative surgical site infections requiring reoperation, and one patient experienced a superficial venous thrombosis, managed nonoperatively. Overall complication rates following Latarjet also vary widely in the literature, with pooled analyses ranging from approximately 6.8% to 30%, and graft- and hardware-related complications most frequently described.^2^^,^^3^^,^^10^^,^^15^ The primary objective of the present study, however, was to evaluate early clinically documented neurological complications rather than radiographic outcomes. Graft union and resorption were not systematically assessed as study endpoints. Within the 6-month follow-up period, however, no patient underwent revision surgery due to radiographic concerns such as graft failure or evident screw malposition.

Although the Latarjet procedure, whether performed arthroscopic or open, is a technically demanding procedure, prior work has demonstrated that complication rates decrease substantially with surgeon experience, from 14.7% in the first half of a surgeon's practice to 3.9% in the second half.^1^ As mentioned, in this series, the low complication rate likely reflects a combination of the surgeon's familiarity with the procedure and the standardized approach utilized. Regardless of experience level, our findings indicate that careful technical execution can minimize complications in the open Latarjet procedure and that previously published high complication rates should not necessarily deter surgeons from selecting to perform a Latarjet when making treatment decisions for anterior shoulder instability.

While this study includes a large consecutive cohort, several limitations must be acknowledged. First, the retrospective design and reliance on chart review introduce the possibility of under-reporting minor or transient neurological symptoms. Early neurapraxias that resolved before the first routine post-operative assessment at four weeks may not have been captured. Furthermore, electromyography was not performed systematically and was reserved for patients with clinically evident deficits. Therefore, subclinical neurapraxias may have gone undetected, and as a result, the true incidence of transient or subclinical neurological events may be underestimated. Second, this study reflects the experience of a single, fellowship-trained, high-volume shoulder surgeon practicing in a specialized setting. Surgeon experience, technical standardization, and procedural volume likely influence complication rates. Consequently, the findings should not be interpreted as representative of general practice, and external validity is inherently limited. The reported complication profile may reflect outcomes achievable in a high-volume environment rather than broadly generalizable risk estimates. Third, 12 patients (6.7%) were excluded due to the absence of post-operative documentation within six months. Although a comprehensive review of institutional and affiliated hospital records was performed, complications managed outside this network may not have been captured, representing a potential source of underestimation. Finally, the absence of a comparative cohort and the retrospective design limit the ability to determine which specific technical factors may have contributed to the low rate of clinically evident neurological complications. Causal inferences regarding individual elements of the surgical technique therefore cannot be established.

## Conclusion

In this single-surgeon consecutive series of primary open Latarjet procedures, early post-operative nerve complications were infrequent, and the overall clinical complication rate was low. These findings reflect outcomes achieved in patients without prior open surgery, utilizing a standardized technique, and should be interpreted in the context of surgeon experience and volume.

## Disclaimers:

Funding: No funding was disclosed by the authors.

Conflicts of interest: Dr. Werthel receives royalties for shoulder arthroplasty implants and is a consultant for Stryker.

Dr. Schoch receives royalties from Advita, Responsive Arthroscopy, Innomed, Buxton and is a consultant for Arthrex.

Any additional authors, their immediate families, and any research foundations with which they are affiliated have not received any financial payments or other benefits from any commercial entity related to the subject of this article.
